# HIV-Associated Neurocognitive Disorders: A Global Perspective

**DOI:** 10.1017/S1355617717001102

**Published:** 2017-10

**Authors:** Rowan Saloner, Lucette A. Cysique

**Affiliations:** 1 The HIV Neurobehavioral Research Program (HNRP), Department of Psychiatry, University of California, San Diego, San Diego, California; 2 Joint Doctoral Program in Clinical Psychology, San Diego State University/University of California, San Diego, San Diego, California; 3 School of Medical Sciences, Faculty of Medicine, The University of New South Wales, Sydney, NSW; 4 Neuroscience Research Australia, Barker Street, Randwick, NSW; 5 Neuroscience Program and Peter Duncan Neurosciences Unit St. Vincent’s Hospital Centre for Applied Medical Research Centre, and departments of Neurology and HIV St. Vincent’s Hospital Sydney, NSW

**Keywords:** HAND, HIV, AIDS, Neuropsychological functions, Review, Cognition, International

## Abstract

The present review on HIV-associated neurocognitive disorders (HAND) provides a worldwide overview of studies that have investigated the rate and neuropsychological (NP) profile of HAND research since the inception of the 2007 HAND diagnostic nomenclature. In the first part, the review highlights some of the current controversies around HAND prevalence rates. In the second part, the review critically assesses some solutions to move the field forward. In the third part, we present the cross-sectional NP profile in non-Western HIV+ cohorts and in relation to Western cohorts’ findings. The adopted global perspective highlights the successful expansion of NP studies in HIV infection to culturally diverse low- to medium-income countries with high HIV burden. These studies have produced interestingly similar rates of HAND whether patients were naïve or treated and/or virally suppressed compared to the rich income countries where the NP research in NeuroHIV has originated. The perspective also demonstrates that globally, the group which is the most representative of the HIV epidemic, and thus at risk for HAND are persons with chronic HIV infection and survivors of past immunosuppression, while in relative terms, those who have been treated early with long-term viral suppression represent a minority. In the last part, we present a review of the naturalistic longitudinal NP global studies in HIV+cohorts, discuss the role of longitudinal design in solving issues around the question of asymptomatic neurocognitive impairment, and the question of biomarker discovery. Finally, we conclude by calling for greater methods and data harmonization at a global level. (*JINS*, 2017, *23*, 860–869)

## INTRODUCTION

Thirty years after the earliest report of HIV-related neurocognitive complications (Grant et al., 1987), and despite major changes in the clinical presentation of the disease since the introduction of combined antiretroviral therapy (cART), the neurological complications of HIV-infection remain significant, especially when we consider disease burden at a global level.

There is now a consensus that the severity of HIV-associated neurocognitive disorders (HAND) neuropsychological (NP) profile is milder based primarily on U.S. and Australian data (Cysique, Maruff, & Brew, 2004; Heaton et al., 2011), and more recently on global data as reviewed further below. Epidemiological figures show that HIV-associated dementia (HAD) is now relatively rare (2–4%) (McArthur, 2004). In contrast, milder forms of the disease (mild to moderate level of global neurocognitive impairment not severe enough to be characterized as dementia) remain fairly common (prevalence varies between 30% without AIDS and 50% in HIV+ adults with current/historical AIDS and in the context of no overt NP confounds) (Cysique et al., 2014; Heaton, Clifford, et al., 2010; Munoz-Moreno et al., 2014; Robertson et al., 2014; Robertson, Smurzynski, et al., 2007; Sacktor et al., 2016; Tozzi et al., 2005).

In stably virally suppressed cohorts or HIV-infected (HIV+) individuals treated early, the HAND prevalence rate varies between 20% and 30% (Bloch et al., 2016; Crum-Cianflone et al., 2013; De Francesco et al., 2016; McDonnell et al., 2014; Wright et al., 2015). There is, however, some debate concerning these results because two studies have found no significant difference between impairment rates in virally suppressed cases compared to their HIV- counterparts (Crum-Cianflone et al., 2013; McDonnell et al., 2014). Also, one outlier study found a HAND prevalence rate of >70% in virally suppressed HIV+ individuals with no NP confounds (Simioni et al., 2010).

When considering this pool of studies in the United States, Western Europe, and Australia, we can unequivocally observe that the outlier studies are in the minority (whether they detected low and non-significantly different impairment rates compared to controls or high impairment rates in their HIV+ cohorts), yet their impact has been major. Inexplicably, some of these outlier studies have received more attention outside the NeuroHIV field as proof that HAND may not actually exist and that most of the impairment detected is *first and foremost* due to alcohol/drug use, uncontrolled neurological or psychiatric disorders, low education or being poor, female, and black. This interpretation, however, concentrates on the studies conducted in the Western centers. By taking a more global perspective, the review will highlight a more nuanced interpretation.

This global perspective will help the reader to form a critical overview of the current debate around prevalence rates and possible solutions. The global perspective also highlights one of the major achievements of the NeuroHIV research community: the successful expansion of NP studies in HIV infection to culturally diverse low- to medium-income countries with high HIV burden. This expansion has demonstrated that globally, the majority of persons with HIV infection are survivors of past immunosuppression, as opposed to early treated virally suppressed persons. Although this situation will change in the future, historically immune-suppressed persons are those who are currently aging, while many of their counterparts have died of AIDS with or without HAD. We are, therefore, currently studying a majority of survivors, who have withstood years of HIV-associated biological insults, with major potential impact on prevalence, incidence and severity and related biomarker research. Early treated patients are younger, but as they age, they could be representative of “less biologically resilient” individuals.

## PREVALENCE CONTROVERSIES: ISSUES OF DIAGNOSTIC ACCURACY

Part of the validity of the Frascati HAND diagnostic criteria (Antinori et al., 2007) has been recently challenged (Gisslen, Price, & Nilsson, 2011; Nightingale et al., 2014). While there is general agreement that NP testing is probably accurate in determining moderate to severe forms of HAND, researchers disagree over the criterion validity of NP testing in accurately identifying a clinically meaningful level of mild neurocognitive impairement, and in delineating asymptomatic neurocognitive impairment (ANI) from impairment resulting from confounding factors. Because of this, several studies have resorted to only including mild neurocognitive disorder (MND) and HAD cases, which can be useful at the level of primary care (McCombe, Vivithanaporn, Gill, & Power, 2013). But for biomarker discovery and prognostic value, the *a priori* exclusion of ANI is an issue, as this mild level of HAND is predictive of further cognitive decline (Grant et al., 2014; Sacktor et al., 2016).

There is empirical evidence that the Frascati criteria recommending at least a minus one standard deviation (−1 *SD*) on two cognitive domains, or 16% *low NP performance threshold* in an HIV- control sample optimally balances specificity and sensitivity for mild HAND detection (Taylor & Heaton, 2001). Yet researchers who focus on this number at face value interpret that HAND rates are systematically over-estimated (Gisslen et al., 2011; Nightingale et al., 2014). While some researchers contend that a -1 *SD*, as opposed to a proposed −1.5 or −2 *SD*, impairment threshold reflects the influence of confounding factors (e.g., psychiatric disorders that are common in HIV population) on rates of ANI, proponents of the −1 *SD* threshold point to data demonstrating relationships between objective markers of HIV brain involvement (e.g., neuropathology), and mild NP impairment at −1 *SD* (Cherner et al., 2002; Masliah et al., 1997; Moore et al., 2006).

Furthermore, there is substantial cumulative evidence that a cut-off at −1.5 *SD* or −2 *SD* will indisputably miss patients who have disease (Cherner et al., 2007; Grant et al., 2014; Tierney et al., 2017). Importantly, low NP performance in an HIV- sample is not the *exact equivalent* of impaired NP performance in a clinical sample. A recent review (Gates & Cysique, 2016) has attempted to clarify this issue where it was demonstrated that the recommended −1 *SD* in two cognitive domains *low NP performance threshold* in an HIV- control sample yielded a 5% misclassification rate for HAND in the demographically comparable HIV+ sample, and not a 16% rate. This result is associated with two factors: higher correlations between NP tests measures as a function of greater impairment, and the selection of test batteries that focus on detecting HIV-related cognitive impairment but not general cognitive impairment.

Adding to the misunderstanding is the fact that some researchers have computed the Frascati criteria incorrectly when conducting comparisons between the Frascati criteria and potential new ones (Su et al., 2015). Computational errors may also explain why some studies found unusual discrepancies between the cognitive rating and the global deficit score methods (McDonnell et al., 2014), despite the virtual equivalence between the two methods (Blackstone, Moore, Franklin, et al., 2012). Furthermore, in several instances, researchers assessing the robustness of the Frascati criteria in small test batteries have not modified—as required in this context (Ingraham & Aiken, 1996; Kamminga et al., 2017) —the expected low performance rate in their HIV- control sample to meet the recommended 16% low-performance threshold. Failure to modify the expected threshold for impairment in small batteries by either using a stricter cut-off (Kamminga et al., 2017), or/and requiring impairment in only one task (Ingraham & Aiken, 1996) will produce unexpectedly high rates of low performance in HIV- (McDonnell et al., 2014). Finally, some reports have not used appropriate normative data (Simioni et al., 2010) or have used control samples that diverged substantially from clinical samples in terms of some socio-demographics (McDonnell et al., 2014).

## PREVALENCE CONTROVERSIES: THE ROLE OF NP CONFOUNDS ON HAND PREVALENCE

The HIV epidemic is characterized by situations that make the detection of HAND in very specific populations or demographic categories challenging. For example, the study by Crum-Cianflone et al. (2013) evaluated NP functions in 200 HIV+ and 50 demographically comparable HIV- military beneficiaries. The HIV+ sample was relatively healthy (HIV duration=5 years; 64% on cART initiated; median CD4 of 333 cells/mm^3^). The overall impairment rate was found to be 19% in the HIV+ group and surprisingly 30% in the HIV- group. While the level of impairment in the HIV+ group is akin to another study in patients with high CD4 cell counts and cART initiation always above CD4 of 350 cells/mm^3^ (despite different methods it was 20%) (Wright et al., 2015), the rate of impairment in the HIV- sample was very unusual and akin to a clinical population with a history of traumatic brain injury (Rabinowitz et al., 2015), condition that is prevalent in the U.S. military (Helmick et al., 2015).

The largest ever study of HIV- and HIV+ women (Maki et al., 2015) showed an overall small effect of HIV on cognition (demographically adjusted Cohen’s *d*<.20 ) in a cohort composed of HIV+ women where 30% had AIDS, 53% were virally undetectable and 35% were sub-optimally adherent. In relative terms, the effect of reading achievement, age, years of education, and racial/ethnicity category had a much larger explanatory value with respect to NP performance. However, the effect of HIV disease biomarkers (viral detection, lower CD4 and AIDS) on cognition was detected yet masked at the group level due to a dramatic combination of socio-economic burdens (poverty, childhood trauma, domestic violence) that negatively and independently impacted cognition in both HIV- and HIV+ women, particularly for learning, memory, and psychomotor speed (Rubin et al., 2016). Interestingly, the longitudinal follow-up of this cohort can be found in Rubin et al., (2017). An editorial highlighted the main finding which was that cognitive decline was greater in HIV+ women whether virally suppressed or not compared to their HIV- counterparts and above and beyond the effect of confounds (Cysique & Becker, 2017).

## THEMES TO CONSIDER IN THE POTENTIAL UPDATE OF THE FRASCATI CRITERIA

New indexes may be considered for improving the detection of clinically meaningful ANI/MND, but they will need further study to assess their validity. Investigators of the POPPY studies have proposed other statistical indexes. However, this work is only based on the CogState battery (De Francesco et al., 2016). Any CogState cognitive domain grouping would require some validation as this battery is different from standard NP testing (Cysique, Maruff, Darby, & Brew, 2006; Kamminga et al., 2017), while a global score as opposed to cognitive domain ratings is best to quantify impairment when using this battery (Kamminga et al., 2017). One proposed method (also used by Su et al., 2015) is the multivariate normative comparison (MNC), which has been developed to control for family-wise error in large NP test batteries (Huizenga, Agelink van Rentergem, Grasman, Muslimovic, & Schmand, 2016). Recent improvement in the MNC method shows that individual cases need to be compared to normal age, education and gender matched cases, and not the entire normative sample (Agelink van Rentergem, Murre, & Huizenga, 2017). To the best of our knowledge, this requirement has not been conducted in NeuroHIV studies which used this method. Also, the MNC validity to detect mild cognitive impairment in non-HIV populations has not been tested. The other method propsed by POPPY investigators (Underwood, Leech, Winston, Sabin, & De Francesco, 2017) is the Malahanobis distance (Crawford & Allan, 1994), which *a priori* takes into consideration the test correlation within a test battery while the Frascati criteria do not, and the Global Deficit Score [GDS, (Carey et al., 2004)] does, but not to the same extent (Gates & Cysique, 2016). Similar to the GDS, however, the Malanobis distance needs to be thresholded, so the issue of how to accurately detect clinically meaningful forms of mild cognitive impairment remains.

The exclusion of neuropsychiatric symptoms from the Frascati criteria is problematic because it is well known that alteration to striato-frontal circuits affect emotional regulation and motivation (Cole, Castellon, et al., 2007). Therefore, some neuropsychiatric symptoms prevalent in HIV+ persons, including apathy, irritability (Watkins & Treisman, 2012), anxiety (Brandt et al., 2017), possible treatment-resistant depression (Cysique, Dermody, Carr, Brew, & Teesson, 2016), could represent progressing HIV-related brain injury (Cysique & Brew, in press). Furthermore, in women and ethnically diverse cohorts, it appears that major depressive disorder is more often associated with HAND (Fellows, Byrd, Morgello, & Manhattan, 2013), and is more often predictive of cognitive decline (Heaton et al., 2015) than in less diverse male cohorts (Cysique et al., 2007). Such subtle divergences in the expression of a neurological and neuropsychiatric disease should be expected and not systematically interpreted as evidence that “ethnic minorities and women are a problem” or that their disease expression is not the “real” one. Historically, symptoms that were later accepted as part of the CDC AIDS definitions were not initially recognized in African-American women back in the 70s because their symptoms did not correspond to the typical presentation of AIDS in men (The ACT UP/New York Women and AIDS Book Group, 1990). Further illustrating this complexity, a recent study from Nigeria showed that higher plasma levels of HIV driving activation of circulating monocytes may be more explanatory of HAND in women than in men (Royal et al., 2016).

In a potentially new formulation of the Frascati criteria, the question of impact on everyday functioning would have to be considered carefully. The assessment of current everyday functioning primarily relies on patients’ self-insight (Chiao et al., 2013). This is less of an issue in high-functioning persons with mild HAND, who show correlation between insight into their cognitive difficulties and their actual level of cognitive impairment (Cysique et al., 2014); although depressed HIV+ patients typically report more cognitive symptoms than non-depressed patients, and sometimes without any evidence of objective cognitive deficits (Carter, Rourke, Murji, Shore, & Rourke, 2003).

However, as soon as the impairment is moderate, especially in a fronto-striatal disease such as HAND (Thames et al., 2011), insight can be impaired enough that the self-assessment of everyday functioning is likely to be inaccurate (Blackstone, Moore, Heaton, et al., 2012). Therefore, modifying the diagnostic Frascati criteria to require impact on everyday functioning as in the current DSM-5 criteria (American Psychiatric Association, 2013) could have negative consequences by over-representing depressed, while those with lower self-insight and frontal symptoms would be systematically excluded. Recent advances have seen the development of several performance-based activities of daily living assessments which overcomes the issue of self-report (Blackstone, Moore, Heaton, et al., 2012; Sheppard, Woods, & Verduzco, 2017), including some that are Internet based (Woods et al., 2017). More work is needed to assess their construct validity as real-life indicators independent of cognitive functioning.

Further improvement in diagnostic validity will come not from any artificial reduction in the capacity of the criteria to detect baseline mild HAND, but by working toward the identification of biomarkers that will aid in determining if current HAND (including ANI) represent an active brain pathology or a static burnt out process (Brew, 2004). This could be achieved by combining diagnostic criteria that determine if HIV+ patients have, within a clinically relevant period, stable, progressing, or incident cognitive decline (Gott, Gates, Dermody, Brew, & Cysique, 2017; Tierney et al., 2017) against selected biomarkers. The Frascati criteria could be improved by determining guidelines for cognitive decline (Gates & Cysique, 2016; Tierney et al., 2017). These nomenclature changes would be similar to the current DSM-5 criteria for HAND, but with an improved formalization of cognitive impairment and decline (Tierney et al., 2017). Such an update would reduce the weight of any diagnosis on a cross-sectional assessment and confirm its value longitudinally, enhancing the criteria for biomarker discovery, therapeutic and clinical management, as well as selection in trials.

## CROSS-SECTIONAL NEUROPSYCHOLOGICAL PROFILE: GLOBAL FINDINGS IN NON-WESTERN COHORTS IN RELATION TO WESTERN COHORTS

The past decade has witnessed a notable expansion of comprehensive NP studies to Sub-Saharan Africa (Buch et al., 2016), Asia (Joseph et al., 2013; Ku et al., 2014), Eastern Europe (Joseph et al., 2013) and South America (Joseph et al., 2013). These pools of research strongly demonstrate the feasibility of NP studies in culturally and economically diverse non-Western settings using standard NP tests adapted for language and cultural variations. This development represents a remarkable achievement on the part of the global NeuroHIV research community, especially when we consider the paucity of studies that existed in 2009 as reviewed by (Robertson, Liner, & Heaton, 2009). Key to the success of these studies has been the clinical expertise of local researchers, the contribution of collaborative training strategies for local testing personnel (Heaton et al., 2008; Pumpradit et al., 2010), and the commitment of local researchers to the recognition of HAND as a significant health issue.

These studies variously assess NP functions in antiretroviral naïve patients, patients starting treatment, and patients who are stable on treatment where the vast majority had experienced immunosuppression. In untreated cohorts, and as expected, HAD prevalence rate is elevated [e.g., 25.4% in (Joska et al., 2011); 39% in (Sacktor et al., 2013]), similar to pre-cART Western cohorts. The profile of HAD in untreated non-Western samples is identical to that of original reports in the United States, with a subcortical profile dominated by slowing of information processing, motor deficits, working memory/attention deficits, learning and retrieval memory impairment, verbal fluency deficits, and executive dysfunctions (Gupta et al., 2007; Robertson, Nakasujja, et al., 2007; Yepthomi et al., 2006). Although the effect of education on performance is often larger than in Western cohorts (Joska et al., 2012), this is probably due to a wider range of educational experience (Gupta et al., 2011). The use of appropriate controls, fairly comprehensive NP test batteries, and the rigorous application of the Frascati criteria have ensured an excellent detection of HAND (Gupta et al., 2007; Heaton et al., 2008). However, tests requiring high levels of abstraction are probably best avoided among participants with very low levels of educations (Heaton et al., 2008), and reserved for participants with at least secondary school level (Gupta et al., 2014). Finally, similar to North-American cohorts, patients with AIDS (*vs*. non-AIDS) show increased levels of cognitive dysfunction (Kanmogne et al., 2010).

Several studies (Ghate et al., 2015; Joska et al., 2012; Robertson et al., 2012; Sacktor et al., 2013; Valcour et al., 2009) demonstrated NP improvement after cART initiation. As a pool, these studies are more representative of an initial cART effect than Western studies because most patients were originally naïve participants, while in Western cohorts many were ART-experienced (Cysique & Brew, 2009). In studies using fairly comprehensive NP testing and a large number of patients with HAD, the domains that showed the most improvement were motor deficits, working memory/attention deficits, verbal fluency deficits, and executive dysfunctions, while improvement in learning/memory and psychomotor speed were either less pronounced or less consistent (Joska et al., 2012; Sacktor et al., 2013). In patients with mild to moderate or moderate levels of impairment (among which few were affected by HAD), most improvement occurred in learning, and to a lesser extent memory and motor functions (Ghate et al., 2015).

In long-term cART-treated cohorts including a majority of HIV+ patients who had initiated cART with a low CD4 count, a recent study in Zambia (Kabuba, Anitha Menon, Franklin, Heaton, & Hestad, 2017) detected 34.6% of HAND. This prevalence rate is not statistically different from North American studies, which have used the same NP methods [(Heaton, Clifford, et al., 2010), including demographically corrected normative standard]. Also ANI is the most common HAND subtype (68%) showing that its *a priori* exclusion would be a major issue for the accurate assessment of HAND’s global burden. Moreover, the study demonstrated medium effect size impairment between controls and HIV+ participants in the domains of executive functioning, speed of information processing, working memory, learning, delayed recall, but not fine motor-coordination.

Another study of cART-treated patients in Botswana (Lawler et al., 2011) detected 37% with HAND. The study showed impairment in all domains tested (learning/memory; verbal fluency, executive functions, psychomotor speed) compared to demographically comparable controls. Of interest, fine motor-coordination was different at only *p*=.04 between groups. This relative insensitivity of the Grooved Pegboard to HIV effects has been found in several other African studies (Akolo et al., 2014; Clifford et al., 2007; Robertson, Nakasujja, et al., 2007). This could be consistent with the Western studies showing less motor-coordination deficits in cART compared to pre-cART cohorts (Cysique et al., 2004; Heaton et al., 2011), except that it has also been found in asymptomatic HIV+ patients in Nigeria that were not on treatment (Akolo et al., 2014).

Future studies dedicated to exploring this issue could perhaps use a more extensive fine-motor assessment, a thorough assessment of demographic factors that may affect manual dexterity (Gupta et al., 2011), and control for any peripheral neuropathy(Fellows et al., 2012). In this regard, focusing assessment on motor functions should also probably be avoided when possible in non-Western cohorts, as it seems to lead to lower than expected rates of HAND (Robertson et al., 2011; Wright et al., 2008) (<15%). Finally, a meta-analysis by (Habib et al., 2013) for sub-Saharan countries which included studies using screening tests and more comprehensive NP testing found that among those on ART for ≥6 months, the estimate for neurocognitive impairment was 30.4%. This is mostly consistent with what is detected in Western cohorts.

In other parts of the world, and in cohorts composed of mostly virally suppressed HIV+ persons tested with comprehensive NP test batteries, impairment rates were found to be around the 25–35% benchmark (Ene et al., 2014; Heaton, Cysique, et al., 2010; Kelly et al., 2014; Ku et al., 2014; Pumpradit et al., 2010). In these studies, as in Western cohorts, ANI again accounts for the majority of diagnosis, MND for approximately a quarter, and HAD is typically present in less than 5%. Slight variation in HAND prevalence rates may be accounted for by differences in past experience of immunosuppression/viral replication, timeliness of cART initiation, duration of stability of current cART, and some portion of confounds if those were not excluded or controlled for.

## LONGITUDINAL NEUROPSYCHOLOGICAL PROFILE: GLOBAL NATURALISTIC STUDIES

This overview of naturalistic longitudinal studies focuses on observational studies and their definitions of cognitive decline, thus exclusive of drug trials. We included 10 naturalistic longitudinal studies of cognitive change with *cognitive decline* investigated as a primary outcome as reported by August 2017, inclusive of a majority of HIV+ participants on stable cART. Seven were conducted in the United States, two in Australia and one in China, highlighting the need for further expansion. Several principal findings can be extracted from those studies: (1) The definition of cognitive decline as an absorbing state (wherein once an individual is defined as having declined this status is permanent) as compared to *studies with multiple follow-up points* (wherein an individual’s status can fluctuate across the duration of the project) yields *apparent* differences in cognitive decline rates that have to be interpreted carefully. Cysique, Maruff, and Brew (2006) defined cognitive decline as a unique endpoint and found 30% of HIV+ participants were defined as decliners at 6–12 month follow-up and 5% at 15–27 months.

In contrast, the large CNS HIV Antiretroviral Therapy Effects Research (CHARTER) study which defined cognitive decline as an absorbing state found an overall 23% cognitive decline rate over the 3-year study follow-up (Heaton et al., 2015). A subsequent project by Cysique et al. (2010) also used an absorbing rate definition and found an overall decline over the 27-month study period of 27%. (2) These three studies used standard global change scores (*standard* in the sense that scores are corrected for practice effect and regression toward the mean) and found fairly similar rates of cognitive decline ranging between ~30% across different cohorts context/nationalities. (3)

Some studies focused on incident HAND. One study (Sheppard et al., 2015) found that HIV infection confers a nearly fivefold risk for developing a neurocognitive disorder over approximately 1 year. The sample was composed of a majority of participants on cART, of which 26% had detectable viral load. Of note, the authors applied a custom-made group practice effect correction based on the sub-sample of patients who had stable performance. They detected that 15.7% of the cohort had incident HAND. Another study (Gott et al., 2017) in a sample of fully virally suppressed patients detected that incident HAND occurred in 7%, progressing HAND in 10%, while 35% has stable HAND over a 18-month period. The study used standard global change scores and combined this information with baseline HAND status. (4)

Studies that assessed deterioration within the various stages of HAND (thus not correcting for practice effect and regression toward the mean) typically found lower rates of cognitive decline. The Multicenter AIDS Cohort Study (MACS) found that across four years of follow-up, 10% of HIV+ individuals progressed to a worse stage of HAND stage (Sacktor et al., 2016). Another study (Brouillette et al., 2016) reassessed the CHARTER sample using a relatively new group trajectory method but without practice effect corrections and found 15% cognitive decline.

Finally, in a more recent study, Vassallo et al. (2017) found 32% of cognitive decline in a group of patients of which approximately a quarter had detectable viral loads. While they used no correction for practice effect, they chose a fairly stringent criterion for degree decline (-2 *SD*) but only in one cognitive domain, potentially explaining the >30% decline rate. (5) Studies that used a restricted number of tests showed inconsistent results, suggesting that as with cross-sectional studies, a minimum of five cognitive domains should be assessed (Antinori et al., 2007). For example, when focusing on verbal memory, one study found worse performance over time in HIV+ individuals compared to HIV-individuals at 1 year follow-up (Seider et al., 2014) and worse in the oldest participants, while another which focused only on two tasks of psychomotor speed and mental flexibility and a large selected group of long-term asymptomatic HIV+ adults reported stable cognitive performance across eight years (Cole, Margolick, et al., 2007). These results from the MACS cohort have never been replicated, probably because particularly healthy individuals were selected and many without any evidence of HAND in the first place. Overall, this overview demonstrates that standardization of the definition of cognitive decline is urgently needed, especially as the HIV population is aging with an increased prevalence of age-related comorbidities (Guaraldi et al., 2011), and risk for brain premature aging (Brew & Cysique, 2017; Goodkin et al., 2017).

## CONCLUSIONS

In conclusion, the review purposefully demonstrates that the expansion of standard NP testing to non-Western centers has been done with a high level of success. When taken globally, it is clear that HAND remains an important concern for HIV+ persons which may worsen as they age. To build on this positive momentum for NeuroHIV research, NP methods and data would need to be better harmonized to better circumscribe HAND and its evolution across the life-span, as well as assist in treatment and intervention development.
